# Isolation and Characterization of an Atypical *Metschnikowia* sp. Strain from the Skin Scraping of a Dermatitis Patient

**DOI:** 10.1371/journal.pone.0156119

**Published:** 2016-06-09

**Authors:** Chee Sian Kuan, Rokiah Ismail, Zhenli Kwan, Su Mei Yew, Siok Koon Yeo, Chai Ling Chan, Yue Fen Toh, Shiang Ling Na, Kok Wei Lee, Chee-Choong Hoh, Wai-Yan Yee, Kee Peng Ng

**Affiliations:** 1 Department of Medical Microbiology, Faculty of Medicine, University of Malaya, Kuala Lumpur, Malaysia; 2 Division of Dermatology, Department of Medicine, Faculty of Medicine, University of Malaya, Kuala Lumpur, Malaysia; 3 School of Biosciences, Taylor’s University Lakeside Campus, Selangor Darul Ehsan, Malaysia; 4 Codon Genomics SB, Selangor Darul Ehsan, Malaysia; Louisiana State University, UNITED STATES

## Abstract

A yeast-like organism was isolated from the skin scraping sample of a stasis dermatitis patient in the Mycology Unit Department of Medical Microbiology, University Malaya Medical Centre (UMMC), Kuala Lumpur, Malaysia. The isolate produced no pigment and was not identifiable using chromogenic agar and API 20C AUX. The fungus was identified as *Metschnikowia* sp. strain UM 1034, which is close to that of *Metschnikowia drosophilae* based on ITS- and D1/D2 domain-based phylogenetic analysis. However, the physiology of the strain was not associated to *M*. *drosophilae*. This pathogen exhibited low sensitivity to all tested azoles, echinocandins, 5-flucytosine and amphotericin B. This study provided insight into *Metschnikowia* sp. strain UM 1034 phenotype profiles using a Biolog phenotypic microarray (PM). The isolate utilized 373 nutrients of 760 nutrient sources and could adapt to a broad range of osmotic and pH environments. To our knowledge, this is the first report of the isolation of *Metschnikowia* non-*pulcherrima* sp. from skin scraping, revealing this rare yeast species as a potential human pathogen that may be misidentified as *Candida* sp. using conventional methods. *Metschnikowia* sp. strain UM 1034 can survive in flexible and diverse environments with a generalist lifestyle.

## Introduction

The genus *Metschnikowia* belongs to the family Metschnikowiaceae, which is characterized by the presence of multilateral budding of vegetative cells and by the production of elongated asci with one or two needle-shaped ascopores [1]. *Metschnikowia* includes water and terrestrial ascomycetous yeast that inhabit flowers, fruits, flower- pollinating insects, and lacewings [2–7]. At present, a total of 35 *Metschnikowia* species have been identified [8]. The D1/D2 domain of large subunit (26S) ribosomal DNA region is sufficiently variable to distinguish all known *Metschnikowia* species [1, 9]. However, the presence of several *Metschnikowia*-related species has caused some mixing taxa in the genus *Metschnikowia* [10].

Evidence have emerged that *Metschnikowia* may act as a potential biocontrol of post-harvest fruit rot and exhibit antagonistic behavior against various food-borne microorganisms and dermatophytes [11–15]. The antimicrobial characteristic of *Metschnikowia* relies on the strain-dependent production of a reddish pigment, pulcherrimin that forms a chelate complex with ferric ions [16–18]. Sipiczki *et al*. [18] proposed that *Metschnikowia* isolates synthesise pigment to inhibit the growth of sensitive microorganisms by sequestration of iron. The intensity of pigmentation was correlated with the antimicrobial activity. The pigment production also enables pigmented strains to survive in the hostile environment, especially in the human host [18].

*Metschnikowia pulcherrima* (anamorphic state: *Candida pulcherrima*) is the most studied *Metschnikowia* species as a colonizer from the skin, vagina, rectum, blood, nasopharynx, and sputum [19–22]. Nevertheless, the fungus is likely to cause onychomycosis, acne neonatorum, diaper dermatitis, and tinea pedis [19, 23]. This species was the only known *Metschnikowia* sp. isolated from humans. However, in 2012, Savine *et al*. [8] recovered a pigment-producing *Metschnikowia* non-*pulcherrima* strain IHEM 25107 from sputum specimen of a leukaemia patient [8]. D1/D2 domain-based phylogeny revealed that the strain IHEM 25107 was not clustered with all known species and proposed it is a novel *Metschnikowia* species. To date, no study has reported the isolation of no pigment producing *Metschnikowia* non-*pulcherrima* strain from a human. In addition, the phenotypic characteristic of this organism represents an unexplored field.

We describe here the first report of an atypical *Metschnikowia* species which was isolated from the skin scraping of a dermatitis patient. This study aim to identify and characterize the novel *Metschnikowia* species genetically and physiologically. The phenotypic response of this strain to different nutrients and environmental stimuli were also evaluated in this study.

## Materials and Methods

### Ethic statement

Approval for this study was obtained from the Medical Ethics Committee of UMMC (reference number: 201511–1879), and photography consent was obtained.

### Clinical history

**Fig 1:**
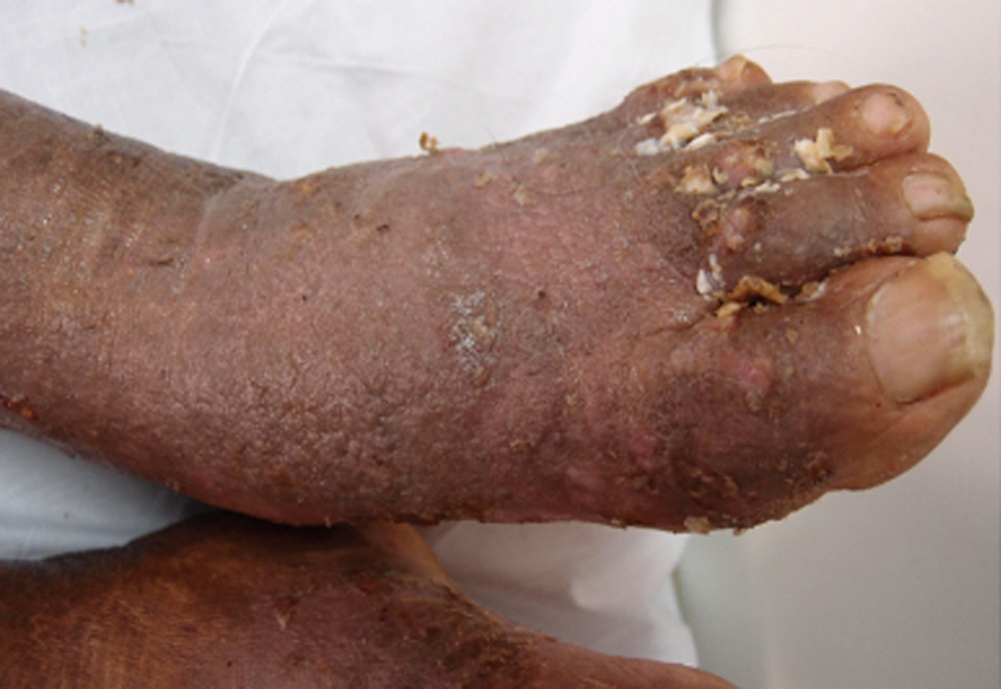
The hyperpigmented, scaly, and lichenified patches over both feet with macerated toe web spaces.

The patient was a 65-year-old man suffering from diabetes mellitus, hypertension, dyslipidaemia, allergic rhinitis with bilateral ethmoidal polyps and gallstone pancreatitis. He was also deaf and mute after childhood meningitis and polio. On 9 September 2014, he was followed-up at the Dermatology Clinic at the UMMC, Kuala Lumpur, Malaysia for stasis dermatitis. He was also treated with topical ketoconazole 2% cream twice daily and oral itraconazole 200 mg twice daily for tinea corporis, tinea cruris, intertrigo and onychomycosis. On 21 September 2014, he was subsequently admitted to the ward due to right lower limb cellulitis secondary to presumed tinea pedis and onychomycosis. On physical examination, there were hyperpigmented, scaly, lichenified patches over both feet; the toe web spaces were also macerated (Fig 1). On 24 September 2014, a skin scraping specimen was collected and sent to the microbiology laboratory for routine microbiological investigations. He was prescribed with intravenous cloxacillin 2 g four times daily for four days, followed by oral cloxacillin 1 g four times daily for a week as well as topical miconazole cream 2% twice daily. The patient was discharged before the microbiological report was obtained. A yeast isolate was isolated from the skin scraping and identified as *Metschnikowia* sp. in October. The patient was readmitted in November 2014 for recurrence of infection at the site of the dermatitis and a 3-day history of fever and painful weepy lesions over his left foot. Physical examination revealed hyperpigmented and lichenified plaques over his lower limbs. The toe web spaces remained macerated with yellowish crusts. The patient was treated with intravenous ampicillin/sulbactam 1.5 g three times daily for three days for likely superimposed bacterial infection causing impetiginisation followed by oral ampicillin/sulbactam 375 mg twice daily for a week, topical 2% miconazole powder to weepy lesions twice daily and topical ketoconazole 2% cream twice daily to dry lesions. He was discharged after five-day treatment.

### Fungal isolate

The skin scraping specimen was processed according to the laboratory’s standard operating procedures (SOP). Direct wet mount was conducted on the clinical specimen by treating with 40% (w/v) potassium hydroxide (KOH). The specimen was first cultured on SDA supplemented with chloramphenicol (0.25 g/mL) for primary isolation of the fungal isolate. Each type of colony grew on the SDA plate were sub-cultured until a pure colony was obtained. Upon successful isolation, the isolate was sub-cultured on SDA (without chloramphenicol) for morphological characterization and subsequent testing. The isolate was maintained by periodic (five days) subculturing on SDA at 25°C with alternate day examination for yeast growth. The isolate was also cultured on SDA at 35°C and 37°C. The chromogenic medium was used for the rapid isolation and identification of clinically important yeast species. Urease test and germ tube test were performed as described by Ng *et al*. [24]. The API 20C AUX yeast identification system (bioMerieux-Vitek, Hazelwood, MO, USA) was used to identify the specific yeast species by reference to the API Analytical Profile Index. The morphological and cultural features of *Metschnikowia* sp. strain UM 1034 were also examined on potato dextrose agar (PDA), corn meal agar (CMA), and V8 agar incubated at 25°C, 35°C, and 37°C for five days. The macroscopic examination was performed to observe colonial characteristics, including the texture and topography. Gram staining was used to observe the microscopic structure of the yeast species grown on SDA, PDA, CMA, and V8 agar.

### *In vitro* antifungal susceptibility

The *in vitro* antifungal susceptibility of *Metschnikowia* sp. strain UM 1034 was determined using the Sensititre YeastOne (TREK Diagnostic System, Cleveland, OH, USA) with a broth microdilution method according to the Clinical and Laboratory Standards Institute (CLSI) M38-A2 guidelines. The isolate was tested against anidulafungin (ANID), amphotericin B (AMB), caspofungin (CAS), fluconazole (FLC), itraconazole (ITC), micafungin (MFG), posaconazole (PSC), and voriconazole (VRC), and 5-flucytosine (5-FC). The concentration gradient for ANID, AMB, CAS, ITC, KTC, PSC, and VRC ranged from 0.002 to 32 μg/mL and the FLC ranged from 0.016 to 256 μg/mL. The minimum inhibitory concentrations (MICs) were examined after 24-hour incubation.

### DNA extraction, PCR and DNA sequencing

The internal transcribed spacer region (ITS), the small subunit of the ribosomal RNA gene (SSU) and the D1/D2 domain of the 26S rRNA gene were used as targets for molecular identification of the yeast isolate. Total DNA extraction, PCR amplification and sequencing were performed as described previously [25, 26]. The ITS, SSU, and D1/D2 domain regions were PCR amplified using primers listed in S1 Table. The ITS, SSU, and D1/D2 domain sequences were searched against the non-redundant (nr) NCBI-nucleotide database using BLASTn program for preliminary identification.

### ITS- and D1/D2 domain-based phylogenetic analysis

As described in previous studies [25, 27], phylogenetic analysis was carried out using ITS (S2 Table) and D1/D2 (S3 Table) nucleotide sequences of UM 1034 isolate together with additional sequences from other *Metschnikowia* species. *Saccharomyces cerevisiae* strain DAOM 216365 was used as an outgroup strain in the phylogenetic analysis. Bayesian tree analyses were performed using MrBayes. Bayesian Markov Chain Monte Carlo (MCMC) analysis was conducted by sampling across the entire general time reversible (GTR) model space. A total of 500,000 and 1,000,000 generations were run for ITS and D1/D2 alignments, respectively with a sampling frequency of 100, and diagnostics were calculated for every 1,000 generations. A burn-in setting of 25% was used to discard the first 2,500 trees.

### Phenotype microarray experiments

Biolog PM analysis (Biolog Inc., Hayward, CA, USA) was employed to examine the metabolic profile of *Metschnikowia* sp. strain UM 1034. Ten MicroPlate panels (PM1 to PM10) including carbon, nitrogen, phosphorus, sulphur, nutrient supplements, peptide nitrogen, osmolytes and pH sources were used in this study. *Metschnikowia* sp. strain UM 1034 was grown on SDA at 25°C for 24 hours. The colonies on SDA were picked and suspended into 15 mL of FF inoculum media (Biolog Inc., Hayward, CA, USA). The cell suspension was adjusted to 62% transmittance at 590 nm using a turbidimeter (Biolog Inc., Hayward, CA, USA). The adjusted cell suspension was inoculated to PM1-10 inoculating fluids (Biolog Inc., Hayward, CA, USA) as indicated in S5 Table. The resultant inoculating fluid (100 μL) was then transferred into each well of PM1-10 microplate and incubated at 25°C for 60 hours in the Omnilog machine (Biolog Inc., Hayward, CA, USA). Negative control measurement was obtained from a well containing water. Data for each assay was reported as an average value from duplicate runs. Heat maps were constructed using Omnilog values ≥ 200,000 recorded over 60 hours.

### Nucleotide sequence accession numbers

The ITS, SSU and D1/D2 domain nucleotide sequences of *Metschnikowia* sp. strain UM 1034 were deposited in GenBank under accession numbers KT186106, KT186108, and KT186107, respectively.

## Results

### Initial microbiological investigations

No fungi elements was observed by a direct microscopic wet mount examination [40% (w/v) potassium hydroxide] on skin scraping specimen. On chromogenic medium, a yeast-like fungus with light purple colour colonies was observed (S1 Fig). The urease test and the germ-tube test were negative. The isolate UM 1034 was presumptively identified as *Candida* species. However, the API 20C AUX yeast identification system revealed that the profile of the isolate was unable to match any identity in the API 20C AUX database.

### Colonial morphology and microscopic examination

**Fig 2:**
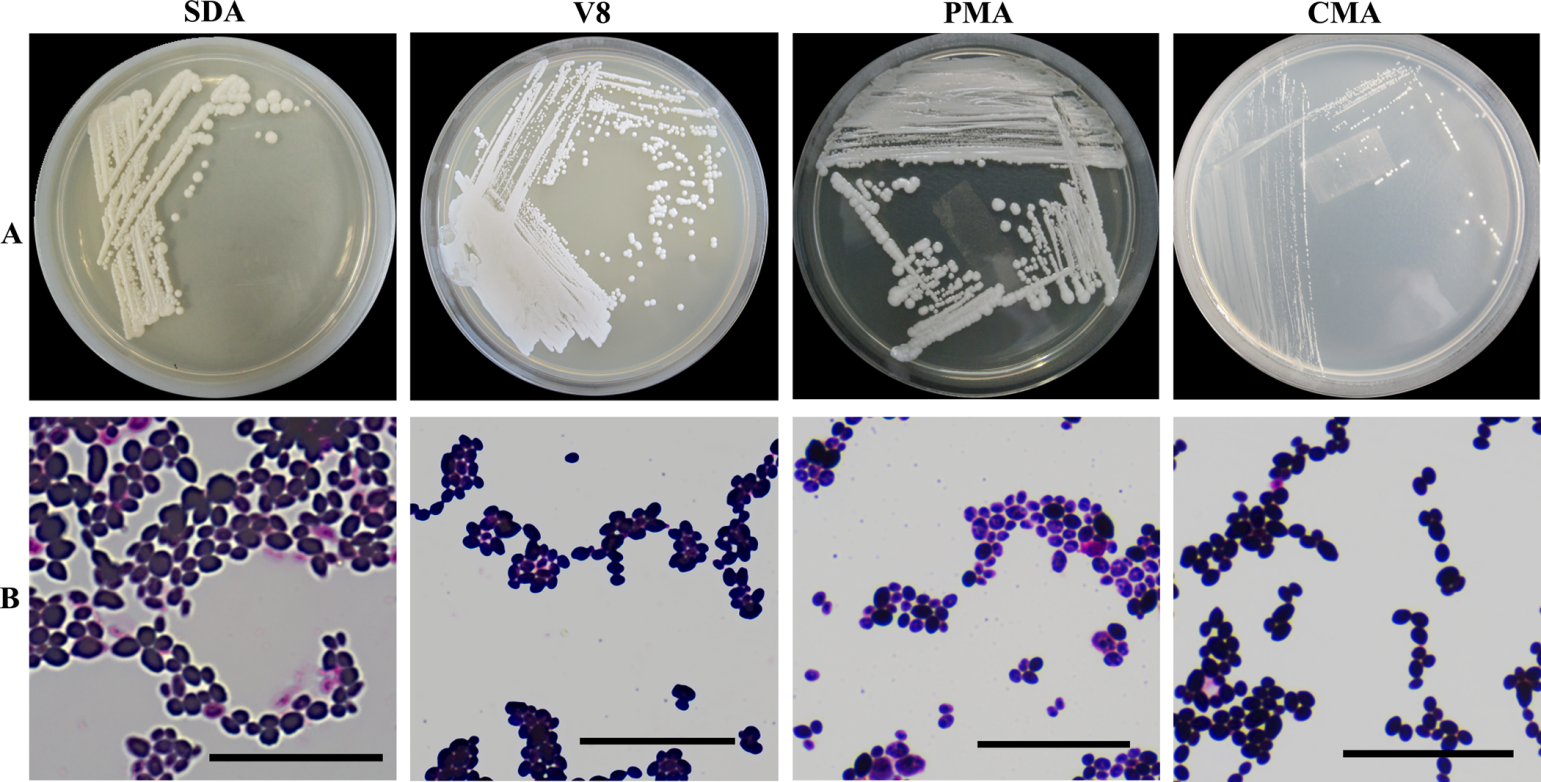
Colonial and microscopic morphology of *Metschnikowia* sp. strain UM 1034. (A) The surface of the colonial morphology of *Metschnikowia* sp. strain UM 1034 after being cultured for five days on SDA, V8, PDA, and CMA at 25°C. (B) Light micrograph showing the micromorphology of *Metschnikowia* sp. strain UM 1034 on SDA, V8, PDA, and CMA (400× magnification, bars 20 μm).

The strain, UM 1034 grew on SDA, PDA, V8 and CMA at 25°C (Fig 2A). No pigment was observed on SDA, PDA, V8 and CMA. The colonies appeared to be white to cream-colored, butyrous and glistening yeast-like on SDA, PDA, and V8. The diameters of the colonies ranged from 2–7 mm, 1–3 mm and 1–5 mm after 5-day incubation on SDA, V8, and PDA, respectively (Fig 2A). The colonies of UM 1034 was observed to be white, tiny, and sparse on CMA. The colonial diameters ranged from 0.3 mm to 3 mm (Fig 2A). The fungus grew faster at 25°C as compared to 35°C. The colonial morphology of the isolate was the same when cultured at 25°C and 35°C. However, no growth was observed at 37°C.

This strain showed a distinct yeast form on SDA, V8, PDA and CMA. Gram-stained smear showed that the yeast-like cells (1–4 × 1–2 μm) were ovoid, ellipsoid or cylindrical shape with budding cells and mother cells in variable sizes (Fig 1C). There were no ascospores produced on 5-day, 14-day, and 28-day incubation at 22°C, 25°C, and 30°C on V8 agar.

### Phylogenetic position

PCR amplification of the ITS, D1/D2 domain, and SSU nucleotide sequences was carried out to confirm the yeast species. By querying ITS, D1/D2 domain, and SSU nucleotide sequences against those deposited in the NCBI-nucleotide database, the isolate showed 97% (367/379) identical to the ITS sequence of *Saccharomycetales* sp. LM376, 99% (538/541) identical to the 26S rDNA D1/D2 sequence of *Metschnikowia* sp. ZB145, and 98% (946/962) identical to the SSU sequence of *Metschnikowia* sp. 11–1129.

**Fig 3:**
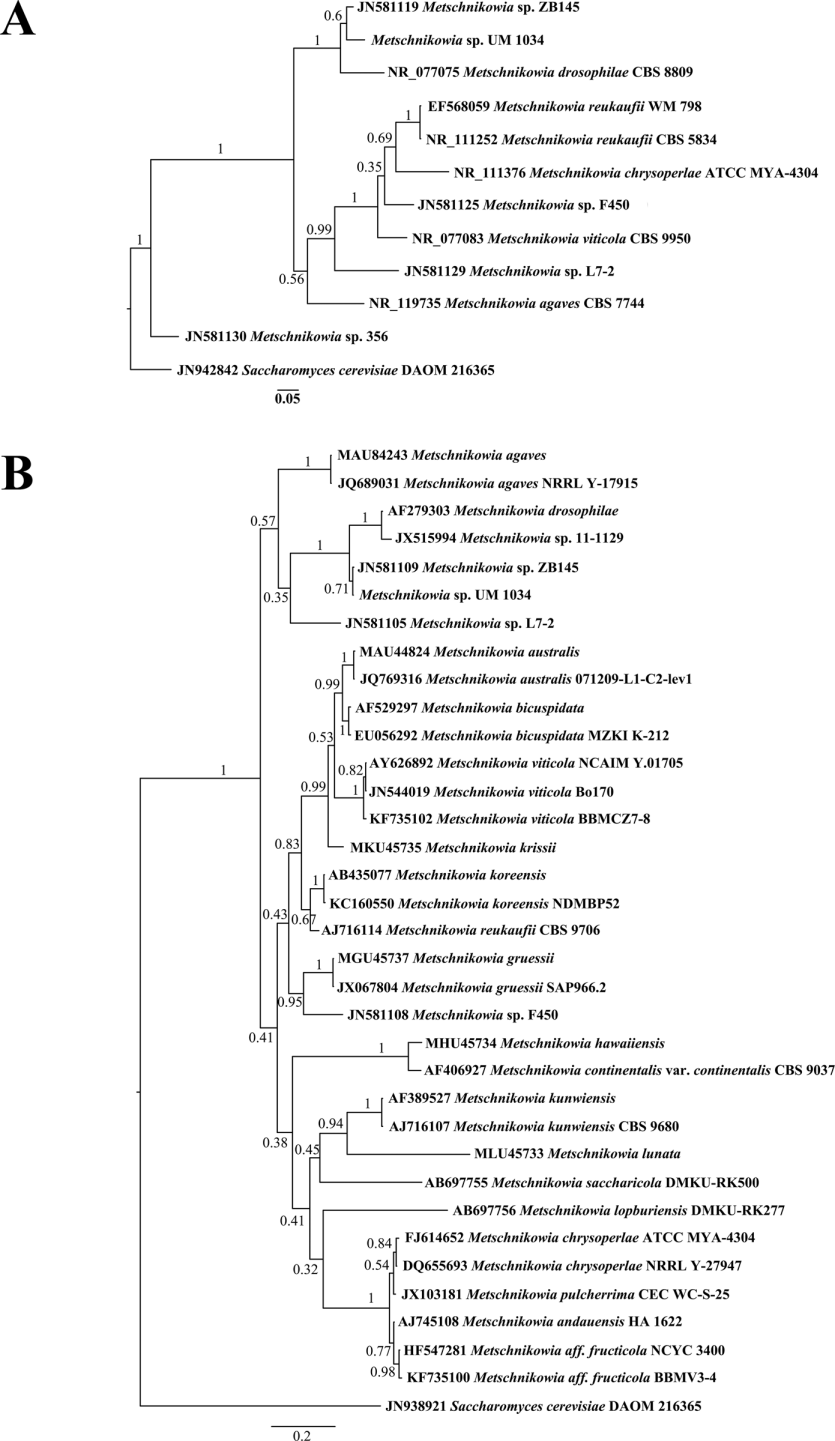
ITS- and D1/D2 domain-based phylogenetic analysis. Bayesian phylogram generated based on (A) ITS and (B) D1/D2 domain sequenced data. The tree is rooted with *Saccharomyces cerevisiae* as outgroup. Numbers on the nodes indicate Bayesian posterior probability based on 100 sampling frequency for a total of 500,000 and 100,000 generations for ITS and D1/D2 alignments, respectively.

The species-level identification of UM 1034 was further confirmed using ITS- and D1/D2 domain-based phylogenetic analysis. The SSU region was excluded in the multilocus phylogenetic analysis due to the limited number of *Metschnikowia* SSU sequences in the NCBI database. As shown in Fig 3A and 3B, the *Metschnikowia* species are well separated. Both ITS- and D1/D2 domain-based phylogenetic analysis revealed that UM 1034 was clustered together with *Metschnikowia* sp. ZB145, distinct from the other described species (Fig 3). However, both the phylogenetic analyses showed that the strain is a sister species to *M*. *drosophilae*. The isolate showed 85.9% similarity to the ITS sequence with the *M*. *drosophilae*. The sequence similarity of the D1/D2 domain between strain UM 1034 and *M*. *drosophilae* was 91.7%. The strain was cautiously labelled as an atypical *Metschnikowia* species by molecular characteristic.

### *In vitro* antifungal susceptibility test

Given that the lack of specific breakpoints for *Metschnikowia* species, we reported only MICs for the isolate and demonstrated clinical breakpoints for the antifungals on *Candida albicans* according to the European Committee on Antimicrobial Susceptibility Testing (EUCAST) and the CLSI (Table 1). Although definitive conclusion cannot be made here, the *Metschnikowia* sp. strain UM 1034 is likely susceptible to all azoles, echinocandins, 5-FC and AMB drugs tested (Table 1).

**Table 1:** Minimum Inhibitory Concentrations (μg/mL) Expressed by *Metschnikowia* sp. Strain UM 1034.

Antifungal drugs	MICs	Methods	Breakpoints (*Candida albicans*)^1^
MFG	0.03 μg/mL	CLSI	≤ 0.25 (S) - > 1 (R)
		EUCAST	≤ 0.03 (S) - > 0.03 (R)
CAS	0.06 μg/mL	CLSI	≤ 25 (S) - > 1 (R)
		EUCAST	ND
5-FC	<0.06 μg/mL	CLSI	≤ 4 (S) - ≥ 32 (R)
		EUCAST	ND
PSC	<0.008 μg/mL	CLSI	ND
		EUCAST	≤ 0.06 (S) - > 0.06 (R)
VRC	<0.008 μg/mL	CLSI	≤ 0.12 (S) - > 0.1 (R)
		EUCAST	≤ 0.12 (S) - > 0.12 (R)
ITC	<0.015 μg/mL	CLSI	ND
		EUCAST	≤ 0.06 (S) - > 0.06 (R)
ANID	0.03 μg/mL	CLSI	≤ 0.25 (S) - > 1 (R)
		EUCAST	≤ 0.03 (S) - > 0.03 (R)
FLC	<0.25 μg/mL	CLSI	≤ 2 (S) - > 8 (R)
		EUCAST	≤ 2 (S) - > 4 (R)
AMB	0.25 μg/mL	CLSI	ND
		EUCAST	≤ 1 (S) - > 1 (R)

^1^S, susceptible; R, resistant; ND, not done

### Nutrient utilization profile of *Metschnikowia* sp. strain UM 1034

Biolog PM was used to screen for the ability of *Metschnikowia* sp. strain UM 1034 to metabolize carbon, nitrogen, phosphorus, sulphur, and other nutrient supplements. *Metschnikowia* sp. strain UM 1034 utilizes nutrients rapidly over 60-hour incubation.

**Fig 4:**
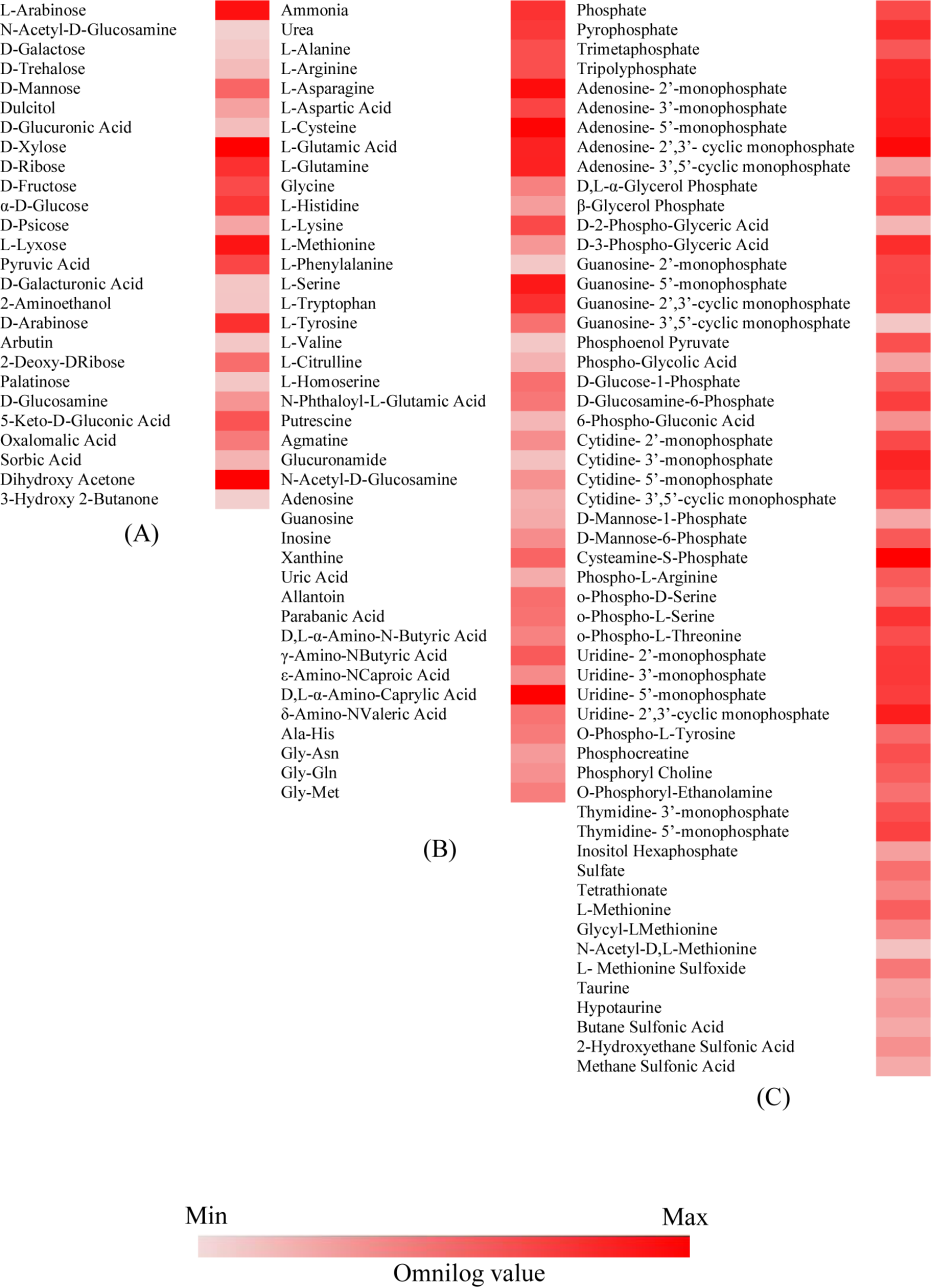
Carbon, nitrogen, phosphorus and sulphur utilization profiles of *Metschnikowia* sp. strain UM 1034. (A) Carbon sources. (B) Nitrogen sources. (C) Phosphorus and sulphur sources. Conditions with a final Omnilog unit (at 60 hours) ≥ 20,000 were incorporated into the heat maps. The growth of *Metschnikowia* sp. strain UM 1034 in a respective substrate over the 60-hour incubation is represented by a color range, as given by the scale bar at the bottom of the figure.

Of the 760 nutrient sources tested, 349 compounds were used by strain UM 1034 (S4 Table). The isolate metabolized 27 of the 190 carbon sources (PM1 and PM2) of the panels (Fig 4A). Most monosaccharides, disaccharides (palatinose and trehalose), and sugar alcohol (dulcitol) could support the growth of the strain. However, the isolate could not utilize any polysaccharide substrates in the panels. Nitrogen utilization pattern for 93 nitrogen substrates (PM3) was also examined (Fig 4B). The analysis revealed that UM 1034 preferred to metabolize organic nitrogens (ammonia and urea), fatty acid (D, L a-Amino-N-Caprylic Acid) and L-amino acids while D-amino acids were the least preferred nitrogen sources. Additionally, the Biolog PM analysis also revealed that UM 1034 can readily metabolize various phosphorus and sulphur sources (65.2% of phosphorus and sulphur sources tested; Fig 4C).

Interestingly, UM 1034 is capable of utilizing all nutritional supplements in PM 5 (S2 Fig). In particular, the strain revealed a greater ability to use 2’-deoxy adenosine, adenosine, adenine, 2’-deoxy inosine, cytosine, 2’-deoxy cytidine, inosine + thiamine, thiamine, butyric acid, D, L-α-hydroxy-butyric acid, and tween 80. Also, we used PM 6, PM 7, and PM 8 to screen a library of di- and tripeptide combinations that cover all 20 amino acids. Fig 4E lists the 136 di- and tripeptides of the 282 peptide combinations utilized by the strain. The most readily utilized of these peptides contained lysine, arginine, and histidine (S3 Fig).

### Chemical sensitivity profile of *Metschnikowia* sp. strain UM 1034

**Fig 5:**
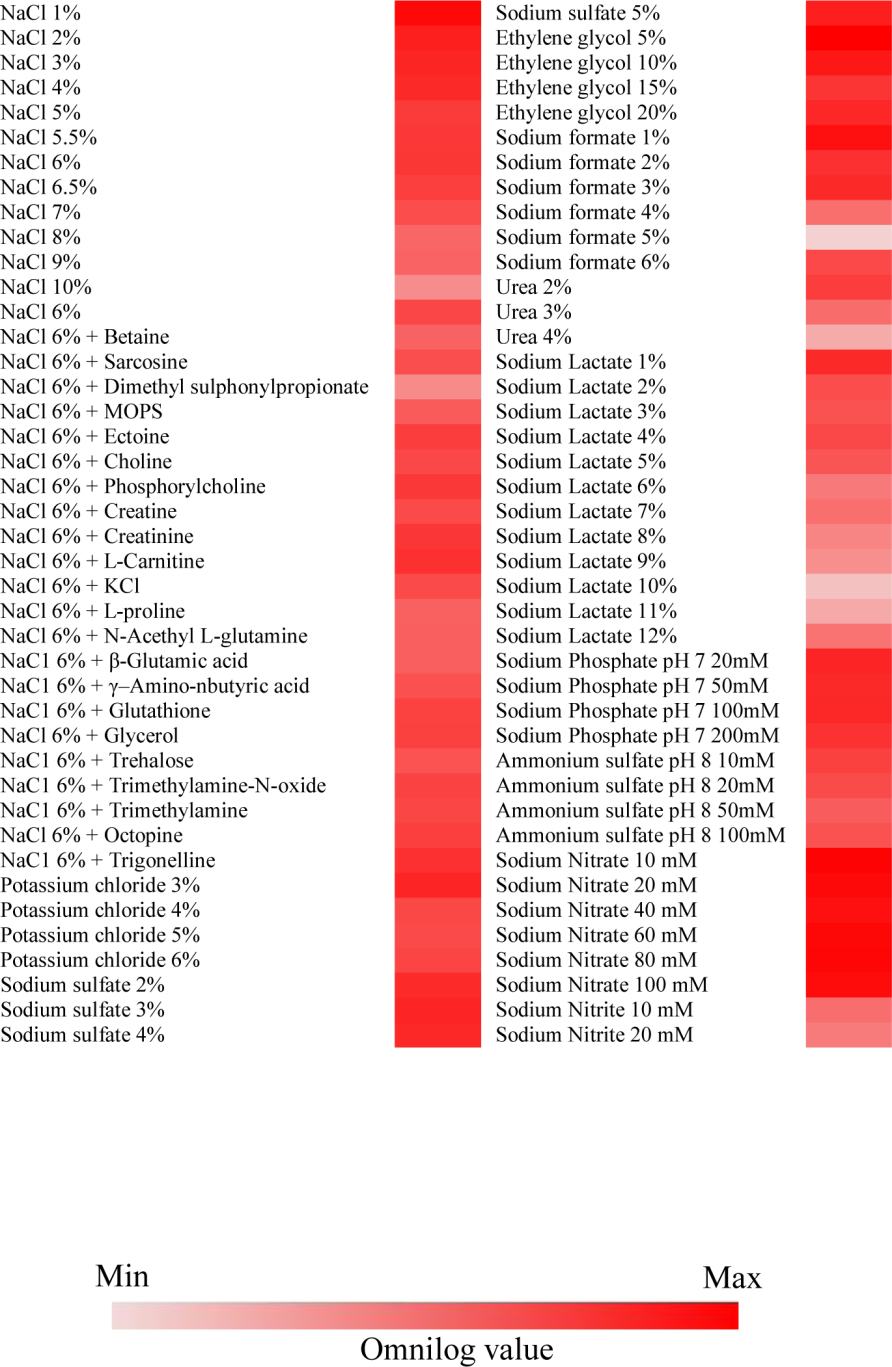
Chemical sensitivity of *Metschnikowia* sp. strain UM 1034. Conditions with a final Omnilog unit (at 60 hours) ≥ 20,000 were incorporated into the heat maps. The growth of *Metschnikowia* sp. strain UM 1034 in a respective substrate over the 60-hour incubation is represented by a color range, as given by the scale bar at the bottom of the figure.

The Biolog PM chemical sensitivity panels (PM 9 and PM 10) were applied to test the sensitivity of UM 1034 to different osmolarity and pH environments. Each chemical sensitivity assay included, at least, four increasing doses of the test chemicals. For sensitivity to osmolytes, UM 1034 was found to be insensitive to numerous sodium salts with detectable growth, including sodium chloride (1–10%), sodium sulfate (2–5%), sodium formate (1–6%), sodium lactate (1–12%), sodium phosphate (20–200 mM), sodium nitrate 10–100 mM), and sodium nitrite (10–20 mM) (Fig 5A). However, PM analysis revealed that two mM or higher concentration of sodium benzoate was toxic to the isolate. The fungus was also found to be able to grow in other osmolytes and ions such as potassium chloride, ethylene glycol, ammonium sulfate, and urea (Fig 5). Furthermore, UM 1034 is capable of growing in pH ranging from strongly acidic to alkaline (pH 3.5 to 10), with maximum growth between pH 4.5 to 7 (S4 Fig).

## Discussion

Superficial skin infections can be caused by dermatophytes, yeast or non-dermatophyte molds [28–30]. The isolation of non-dermatophyte molds from the skin scraping has increased steadily over the last five years [26]. The most formidable challenge is the is the increased emergence of multidrug-resistant strains of fungal isolates, such as *Nigrospora oryzae* [26] and *Pyrenochaeta* sp. [31]. In this study, we isolated and characterized a rare clinical ascomycetous yeast, *Metschnikowia* sp. strain UM 1034, from the skin scraping of a dermatitis patient. Stasis dermatitis is a form of endogenous eczema that occurs on the lower extremities with venous insufficiency. The dermatitis patients usually presented with redness, scaling, itch, erosions, oozing, crusting, blisters and eventually thickened skin. This condition is often complicated by impetiginisation due to secondary infection with *Staphylococcus aureus* and/or coexistent with superficial fungal infections due to maceration and underlying dermatoses. In this case, the patient has diabetes mellitus, which may also predispose to infections due to immunosuppression. Due to the clinical presentation, the patient was treated presumptively with empirical antibiotics for impetiginized dermatitis. Based on the MICs, the yeast is susceptible to azoles and the patient was improved with the treatment.

The genus *Metschnikowia* includes environmental, plant related yeasts. Till now, *M*. *pulcherrima* and *Metschnikowia* sp. strain IHEM 25107 were the only *Metschnikowia* known to have been isolated from human [8, 19–22]. *M*. *pulcherrima* is the only reported pathogen of human and animal in the genus *Metschnikowia* [19, 23, 32]. Both *M*. *pulcherrima* and *Metschnikowia* sp. strain IHEM 25107 contain pulcherrimin pigment which could provide an advantage for the survival in humans [8, 17]. In this study, our molecular and physiological data revealed that the isolate UM 1034 did not match with both *M*. *pulcherrima* and *Metschnikowia* sp. strain IHEM 25107. Also, a typical ascus with ascospore was not observed in this isolate although production of ascospore is a common characteristic of genus *Metschnikowia*. Such observation was in tandem with *Metschnikowia* sp. strain IHEM 25107 which also described the absence of ascospore [8]. However, this isolate lacks the pigment producing ability as compared to both *M*. *pulcherrima* and *Metschnikowia* non-*pulcherrima* strain IHEM 25107. In this case, the pathogenic role of *Metschnikowia* sp. strain UM 1034 is remain questionable. The UM 1034 may be an emerging pathogen to cause real infection or foreign-body reaction because of i.) no other fungi or bacteria was recovered from the skin scraping and ii) the patient was response positive to the azole treatment.

In routine Mycology Laboratory, the identification of isolates is carried out using commercial identification systems that have broad databases. This approach is often combined with conventional phenotypic tests, such as morphological examination, assimilation and biochemical tests. In this study, the UM 1034 was presumptively identified as a typical *Candida* species based on the colonial characteristics, microscopic examination and biochemical test. However, the API 20C AUX yeast identification system failed to identify the yeast species accurately due to the absence of *Metschnikowia* species database in the analytical profile index. These findings led us to perform DNA sequencing and phylogenetic analysis. The ITS- and D1/D2 domain based phylogenetic analyses of strain UM 1034 confirm its placement in the *Metschnikowia* genus. Phylogenetic analyses revealed *Metschnikowia* sp. strain UM 1034 to be a sister species of *M*. *drosophilae*. *Metschnikowia* sp. strain UM 1034 was differentiated phenotypically from *M*. *drosophilae* [33] by its ability to assimilate L-arabinose, D-arabinose, D-ribose as well as its disability to assimilate salicin and glycerol. Kurtzman and Robnett [10] proposed that ascomycetous yeasts with greater than 1% nucleotide variation in the D1/D2 domain of 26S rDNA are probably belonged to separate species. When compared with its closest described relative, UM 1034 differed in the D1/D2 complete sequence by 38 substitutions and 12 gaps from *M*. *drosophilae* (data not shown). UM 1034 differed in the ITS sequence by 52 substitutions and 36 gaps from *M*. *drosophilae* (data not shown). Given these predicted genetic isolation from identified species and phenotypic characteristics, UM 1034 is proposed as a novel *Metschnikowia* species.

In nature, the capability of a microorganism to use various nutrient sources is necessary for survival and adaptation in the composting environment. As our results indicate, the strain UM 1034 has a high capacity to utilize a broad spectrum of nutrient sources particularly noticeable in the case of phosphorus and sulphur sources, as well as nutrient supplements. The comprehensive assessment of nutritional affinity of the strain provides a better understanding of the conditions where it is likely to proliferate, thus allowing the design of interventions to prevent the proliferation of this emerging pathogen. The most significant finding in the data was that UM 1034 is a salt-tolerant yeast, which employs robust osmoadaptation system for utilization of various osmolytes. pH is a vital environment factor affecting pathogenicity; the data showed that UM 1034 can adapt to the wide pH range suggesting that it may harbor an effective pH sensing system to tolerate and survive in hostile environments, like the human hosts.

## Conclusion

We reported the first case of no pigment producing *Metschnikowia* species which was isolated from the skin scraping of a patient with dermatitis. This strain was closely related to *M*. *drosophilae*. Our morphological, molecular, and physiological characteristics proposed that UM 1034 is a novel species of the genus *Metschnikowia*. Although the clinical significance of the isolate in this patient is still uncertain, this strain could be an emerging pathogen associated with dermatitis. In addition, our data shows a comprehensive analysis of nutritional requirements and chemical sensitivities of *Metschnikowia* species. The comprehensive molecular and phenotypic characterization of *Metschnikowia* sp. strain UM 1034 provide a platform to study further its underlying basic biology, lifestyle and potential pathogenicity.

## Supporting Information

S1 Fig Colony morphology of *Metschnikowia* sp. strain UM 1034 on chromogenic agar. (PDF)

S2 Fig Nutritional supplements utilization profiles of *Metschnikowia* sp. strain UM 1034. Conditions with a final Omnilog unit (at 60 hours) ≥ 20,000 were incorporated into the heat maps. The growth of *Metschnikowia* sp. strain UM 1034 in a respective substrate over the 60-hour incubation is represented by a color range, as given by the scale bar at the bottom of the figure.
(PDF)

S3 Fig Peptide nitrogen sources utilization profiles of *Metschnikowia* sp. strain UM 1034. Conditions with a final Omnilog unit (at 60 hours) ≥ 20,000 were incorporated into the heat maps. The growth of *Metschnikowia* sp. strain UM 1034 in a respective substrate over the 60-hour incubation is represented by a color range, as given by the scale bar at the bottom of the figure.
(PDF)

S4 Fig pH sensitivity of *Metschnikowia* sp. strain UM 1034. Conditions with a final Omnilog unit (at 60 hours) ≥ 20,000 were incorporated into the heat maps. The growth of *Metschnikowia* sp. strain UM 1034 in a respective substrate over the 60-hour incubation is represented by a color range, as given by the scale bar at the bottom of the figure.
(PDF)

S1 Table Primers used for PCR amplification. (XLSX)

S2 Table Details of isolates subjected to ITS phylogenetic analysis. (XLSX)

S3 Table Details of isolates subjected to D1/D2 phylogenetic analysis. (XLSX)

S4 Table Auxanographic profile of *Metschnikowia* sp. strain UM 1034. (XLSX)

S5 Table Final concentrations of ingredients in PM inoculating fluids. (XLSX)
