# Supplementary figures and images for: Isolation and Characterization of an Atypical Metschnikowia sp. Strain from the Skin Scraping of a Dermatitis Patient
Source: PLoS One. 2016 Jun 9;11(6):e0156119. doi: 10.1371/journal.pone.0156119 (PMC4900598; doi:10.1371/journal.pone.0156119)

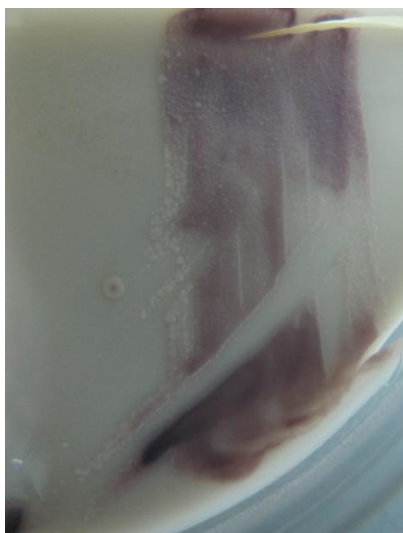

Supplement: S1 Fig — (PDF) [file pone.0156119.s001.pdf]

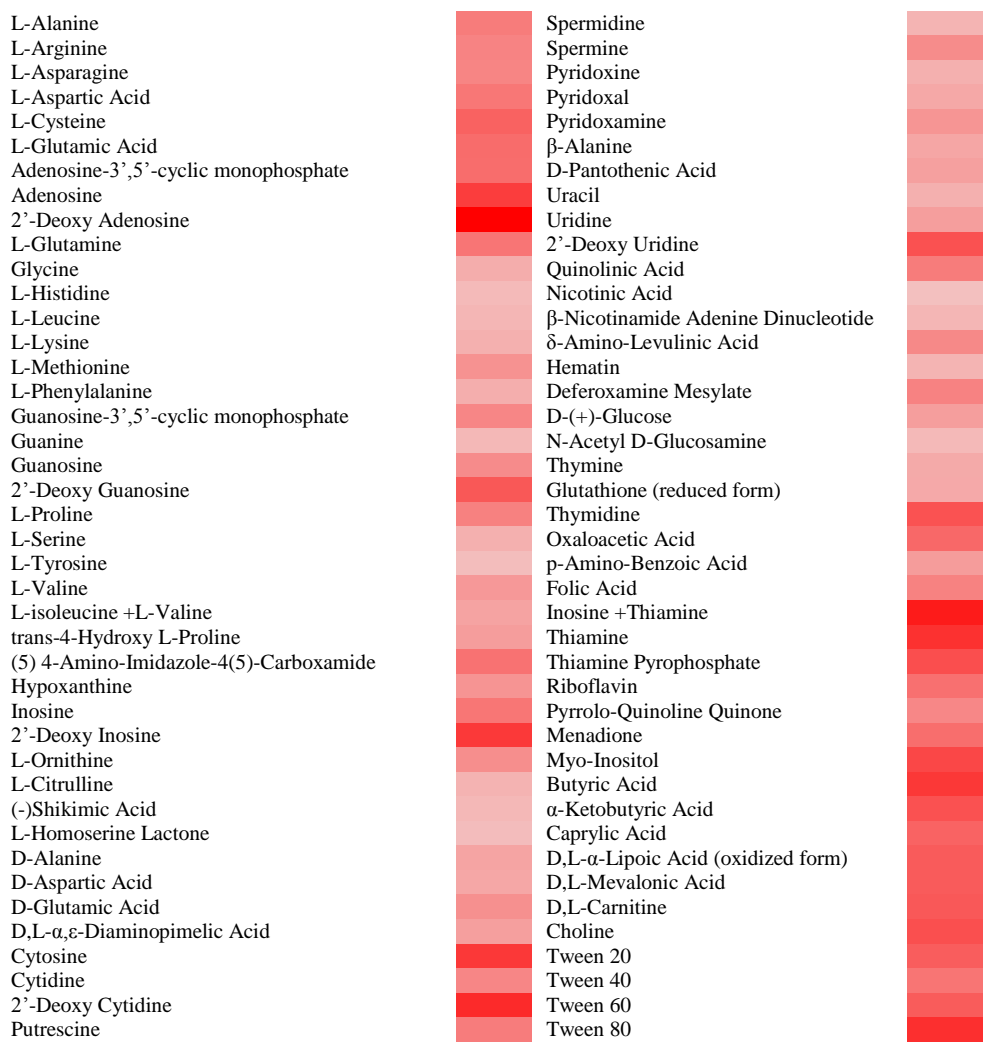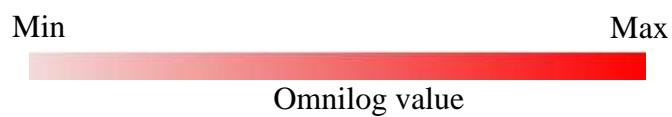

Supplement: S2 Fig — Conditions with a final Omnilog unit (at 60 hours) ≥ 20,000 were incorporated into the heat maps. The growth of Metschnikowia sp. strain UM 1034 in a respective substrate over the 60-hour incubation is represented by a color range, as given by the scale bar at the bottom of the figure. (PDF) [file pone.0156119.s002.pdf]

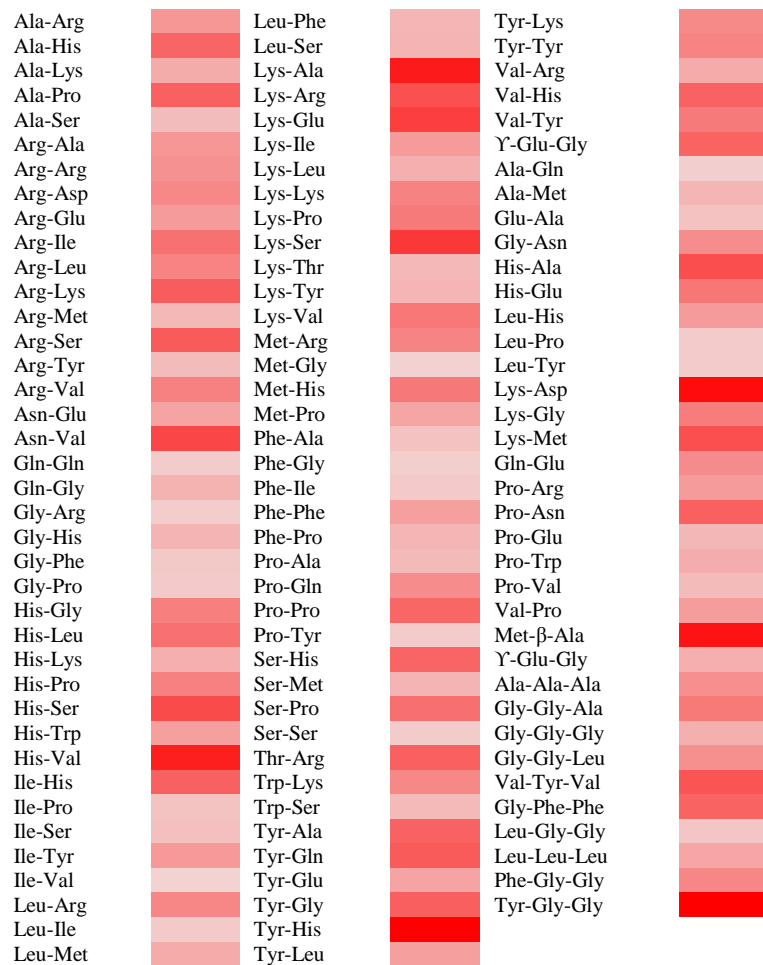

Min

Max

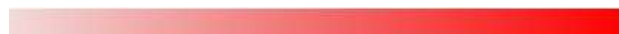

Omnilog value

Supplement: S3 Fig — Conditions with a final Omnilog unit (at 60 hours) ≥ 20,000 were incorporated into the heat maps. The growth of Metschnikowia sp. strain UM 1034 in a respective substrate over the 60-hour incubation is represented by a color range, as given by the scale bar at the bottom of the figure. (PDF) [file pone.0156119.s003.pdf]

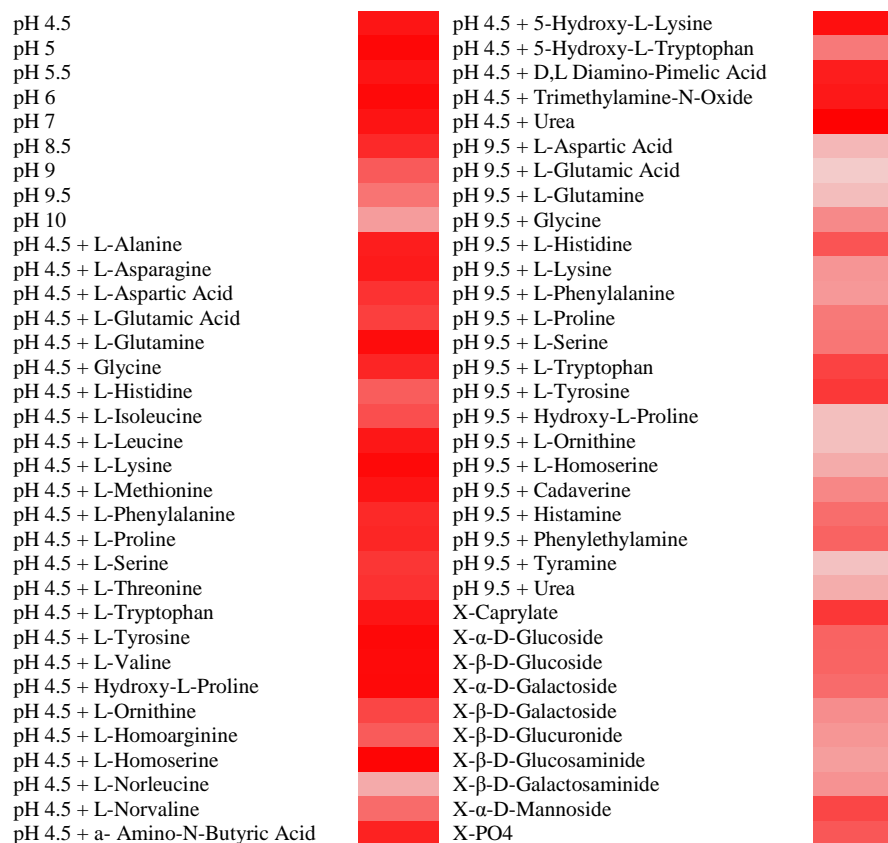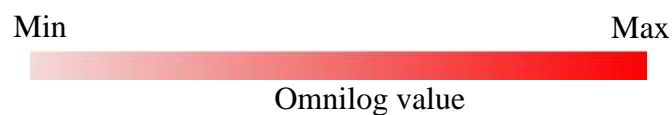

Supplement: S4 Fig — Conditions with a final Omnilog unit (at 60 hours) ≥ 20,000 were incorporated into the heat maps. The growth of Metschnikowia sp. strain UM 1034 in a respective substrate over the 60-hour incubation is represented by a color range, as given by the scale bar at the bottom of the figure. (PDF) [file pone.0156119.s004.pdf]
